# Detection of Ophthalmic Acid in Serum from Acetaminophen-Induced Acute Liver Failure Patients Is More Frequent in Non-Survivors

**DOI:** 10.1371/journal.pone.0139299

**Published:** 2015-09-25

**Authors:** Gurnit Kaur, Elaine M. Leslie, Holly Tillman, William M. Lee, Diane P. Swanlund, Constantine J. Karvellas, US Acute Liver Failure Study Group

**Affiliations:** 1 Department of Laboratory Medicine, Pathology, University of Alberta, Edmonton, Canada; 2 Department of Physiology, University of Alberta, Edmonton, Canada; 3 Department of Public Health Sciences, Medical University of South Carolina, Charleston, South Carolina, United States of America; 4 Division of Digestive and Liver Diseases, Department of Internal Medicine, University of Texas Southwestern Medical Center, Dallas, Texas, United States of America; 5 Divisions of Hepatology and Critical Care Medicine, University of Alberta, Edmonton, Canada; University of Modena & Reggio Emilia, ITALY

## Abstract

**Background/Aim:**

Acetaminophen (APAP) hepatotoxicity is related to the formation of *N*-acetyl-p-benzoquinone imine (NAPQI), which is detoxified through conjugation with reduced glutathione (GSH). Ophthalmic acid (OA) is an analogue of GSH in which cysteine is replaced with 2-aminobutyrate. Metabolomics studies of mice with APAP-induced acute liver failure (APAP-ALF) identified OA as a marker of oxidative stress and hepatic GSH consumption. The aim of the current study was to determine whether OA is detectable in APAP-ALF human patients either early (day 2) or late (day 4) and whether OA levels were associated with in-hospital survival in the absence of liver transplant.

**Methods:**

Serum samples from 130 APAP-ALF patients (82 survivors, 48 non-survivors) were analyzed by liquid chromatography-tandem mass spectrometry (LC-MS/MS) and correlated with clinical data from the United States Acute Liver Failure Study Group (US ALFSG) Registry (2004–2011).

**Results:**

Survivors had significantly lower admission bilirubin (4.2 vs. 5.7 mg/dl) and lactate levels (3.3 vs. 6.5 μmol/l, p<0.05 for all). During the first 7 days of the study, survivors were less likely to require mechanical ventilation (55% vs. 88%), vasopressor support (9.8% vs. 67%) or renal replacement therapy (26% vs. 63%, p< 0.001 for all). Non-survivors were more likely to have detectable OA levels early (31% vs. 15%, p = 0.034) and late (27% vs. 11%, p = 0.02). However there were no significant differences in mean OA levels between non-survivors and survivors (early 0.48 vs. 0.36, late 0.43 vs. 0.37, P > 0.5 for all).

**Conclusion:**

OA was detectable more frequently in APAP-ALF non-survivors but mean OA levels were not associated with survival. The routine clinical administration of *N*-acetyl cysteine could replenish GSH levels and prevent OA production.

## Introduction

Acute liver failure (ALF) is defined by the occurrence of encephalopathy and synthetic dysfunction within 8 weeks of the first symptoms of liver disease [1, 2]. In the United States and United Kingdom, acetaminophen (APAP) overdose is the leading cause of ALF [3, 4]. APAP metabolism occurs in the liver, predominantly through glucuronide and sulfate conjugation. A small amount of the APAP dose is catalyzed by cytochrome P450 2E1 (CYP2E1) to form the reactive intermediate species, *N*-acetyl-p-benzoquinone imine (NAPQI), which can then be detoxified by glutathione (GSH) conjugation. Resulting cysteine and mercapturic acid conjugates can then be eliminated via the urine. If APAP doses are excessive, rapid depletion of GSH from the liver will occur. GSH is an endogenous tripeptide, composed of γ-glutamate, cysteine and glycine, with cysteine being the limiting amino acid. The cysteine can be replaced by 2-aminobutyrate, forming ophthalmic acid (OA), which is effluxed into the systemic circulation from the liver. Treatment of APAP-induced ALF is through the administration of *N*-acetyl cysteine (NAC) therapy. This elevates cellular cysteine levels, allowing for GSH synthesis [5]. Metabolomics studies of mice undergoing APAP induced ALF have identified OA as a marker for oxidative stress and hepatic GSH depletion [6]. The aim of this study was to determine firstly whether the human serum concentrations of OA were detectable in patients with APAP-ALF and secondly whether OA levels were associated with subsequent clinically relevant outcomes (21-day survival) and hence be a potential biomarker in APAP-ALF.

## Materials and Methods

### Study Design

We performed a case-control study of a total of 130 APAP-ALF patients enrolled by the US ALFSG registry between March 2004 and August 2011. The authors’ Institutional Review Board (IRB)/Health research ethics boards of all enrolling US ALFSG sites *(University of Texas Southwestern*, *University of Washington*, *Yale University*, *Medical University of South Carolina*, *University of Alabama*, *University of California Los Angeles*, *University of California San Francisco*, *Northwestern University*, *University of Michigan*, *University of Pennsylvania*, *Virginia Commonwealth University*, *Emory*, *The Ohio State University*, *University of Kansas*, *University of Alberta)* have approved all research and all clinical investigation has been conducted according to the principles expressed in the Declaration of Helsinki. Consent/assent were obtained from all patients/their next of kin for collection of data in the US ALFSG registry and healthy controls. Patient records/information was anonymized and de-identified prior to use in this analysis. Participants who were medically competent provided written informed consent to participate in this study. In cases when patients were unable to provide written consent (critical illness, hepatic encephalopathy) written assent was obtained by the next of kin. Upon regaining capacity, patients were given the option to withdraw written consent. In those cases, data were not included in the registry. Documentation of participant consent/assent is kept in duplicate at individual sites of the US ALFSG (as above). Health research ethics boards/ Institutional review boards at all sites of the US ALFSG have approved this consent procedure (as above).

### Participants

*Inclusion criteria* were: 1) evidence of ALF due to APAP toxicity according to the enrollment criteria for the ALFSG (see operational definitions) and 2) age ≥18 years; and 3) Grade I to IV HE during the first seven days of study admission (West Haven Criteria; see below). *Exclusion criteria* were: 1) Cirrhosis and 2) patients < 18 years.

### Operational Definitions

For the purposes of this study, ALF is defined as International normalized ratio (INR) ≥ 1.5 and HE within the first 26 weeks of liver disease in a patient with an acute hepatic insult [2]. HE grade is defined by the West Haven Criteria (simplified) as follows; grade 1 ~ any alteration in mentation, grade 2 being somnolent or obtunded but easily rousable or presence of asterixis, grade 3 being rousable with difficulty and, grade 4: unresponsive to deep pain [7, 8].

### Data Sources and Collection

Data were collected prospectively as part of the US ALFSG and retrospectively analyzed. Prior to February 2010, each individual site prospectively sent case report forms to the University of Texas Southwestern for entry into a central database. Following this date, individual sites entered data electronically into a central database housed at the ALFSG Data Coordinating Center at the Medical University of South Carolina (Charleston, USA). Registry data assessed in this study included demographics (age, race, sex), etiology of ALF, hematology and biochemistry, requirement for organ support and therapies on day of admission or during first 7 days, and outcome data (21-day survival).

### Solvents and Reagents

HPLC-grade acetonitrile, HPLC-grade methanol, and formic acid were purchased from Fisher Scientific (Fair Lawn, NJ). Water was purified using a Milli-Q water purification system (Millipore, Molsheim, France). OA was purchased from Bachem (Bupendorf, Switzerland). All other chemicals and reagents used were of analytical grade.

### Patient Serum Sample

Patient samples were acquired from the National Institute of Diabetes and Digestive and Kidney Diseases (NIDDK) BioSample Repository. A total of 260 samples from 130 patients that had suffered APAP-induced ALF were analyzed. Patient blood samples were collected on Day 2 and Day 4 from when the patients were admitted into hospital (Day 0). At Day 21, 82 patients were alive and 48 deceased. A total of 25 healthy control serum samples were acquired from the Centre of Excellence for Gastrointestinal Inflammation and Immunity Research (CEGIIR), University of Alberta for comparison of OA levels in serum from APAP-induced ALF patients. Serum samples from four other healthy controls recruited from the University of Alberta were used for preparation of standards (as described below).

### Preparation of Ophthalmic Acid Standards

OA solutions were prepared in water to achieve concentrations ranging from 0.05 μM to 10 μM. The standard curve was made up of the following concentrations: 0.05 μM, 0.1 μM, 0.3 μM, 0.5 μM, 1 μM, 3 μM, 5 μM, 10 μM. Serum samples (48 μl) from healthy human volunteers (with OA levels below the limit of detection) were spiked with these concentrations of OA (2 μl) and processed as described previously, in order to achieve a standard curve [9]. Briefly, OA was extracted from human samples (25 μl) using ice cold methanol (75 μl). Samples were vortexed and centrifuged at 16,000*g* for 20 min. The supernatant was collected and speed vacuumed to dryness. Contents were then dissolved in 50 μl of water and vortexed before analysis. Water (48 μl), was also spiked in an identical manner without extraction to act as quality control. Fresh standard curves were prepared for each analysis.

### UPLC-quadrupole linear ion trap MS conditions

A quadrupole linear ion trap mass spectrometer (4000 QTRAP, Applied Biosystems/MDS SCIEX, Toronto, Canada) was coupled with an Agilent UPLC system (Agilent Technologies, Waldbronn, Germany). The QTRAP-MS conditions were set as previously described [10]. The HSS T3 (C18) column (2.1 mm x 150 mm), with a 1.8 μm particle size was used. The autosampler and the column oven were kept at 4°C and 60°C respectively. The flow rate was 0.35 ml/min and the mobile phase consisted of Solvent A and Solvent B. Solvent A was made up of 0.1% Formic Acid, 2% Acetonitrile and 98% Water. Solvent B was made up of 0.1% Formic Acid, 2% Water and 98% Acetonitrile; gradient elution conditions were set as previously described (New and Chan, 2008). The injection volume was 3 μl. OA was profiled using an *m/z* transition of 290.1 to 161.1. Data were analyzed using Analyst 1.6.2 software (Applied Biosystems).

### Statistical Analysis

Statistical analysis was performed using IBM SPSS version 22 (2014) and SAS version 9.2 (SAS Institute, North Carolina, USA). In the event of missing values, data were not replaced or estimated. Data were analyzed using descriptive statistics to characterize demographics and other clinical variables. Categorical variables were compared using the Chi-square test or Fisher’s exact test (< 5 subjects). For continuous variables, normally distributed variables were reported as means with standard deviations (SD) and compared by Student’s t-test. Non-normally distributed continuous data were reported as medians with inter-quartile ranges (IQR) and compared by Wilcoxon rank sum test. Survival was defined as the dichotomous outcome, alive at 21-days after enrolment into the Registry. A two-sided significance level of <0.05 was used for all comparisons.

## Results

Demographic, biochemical and therapeutic characteristics of all APAP-ALF patients (n = 130) are shown in Table 1. Survivors (n = 82) were significantly younger (mean 36 (SD 13) vs. 42(15) years) than non-survivors (n = 48) with no differences in gender or sex. On study admission (Table 1), hematological indicators were similar apart from median platelet count (survivors 140 vs. controls 116; p = 0.009). Survivors had significantly lower admission bilirubin (4.2 vs. 5.7 mg/dl; p = 0.001), creatinine (1.4 vs. 2.3 mg/dl, p = 0.049) and lactate levels (3.3 vs. 6.5 μmol/l, p = 0.003).

**Table 1:** Demographic, Clinical and Biochemical Parameters in 130 Acetaminophen-induced Acute Liver Failure patients.

	Survivors (n = 82)		Non-survivors (n = 48)		
	N	Number % or median (IQR)	N	Number % or median (IQR)	P value
**Age**	82	36 (13)	48	42 (15)	**0.007**
**Sex (female)**	82	62	48	34	0.55
**Race**	82		48		0.36
White		66		39	
African-American		7		8	
Other		5		1	
**Admission biochemistry**					
White Blood count (x10^9^/L)	81	9.3 (6.4–13.7)	48	9.3 (7.0–13.3)	0.87
Platelet count (x10^9^/L)	81	140 (91–196)	48	116 (61–164)	0.009
Internationalized Ratio (INR)	82	2.7 (1.8–4.3)	46	3.2 (2.0–4.3)	0.19
ALT (IU/L)	79	3346(1920–6894)	48	3211(1246–6355)	0.53
Bilirubin (mg/dl)	79	4.2 (2.3–5.7)	48	5.7 (3.7–8.2)	0.001
pH	70	7.4 (7.3–7.5)	45	7.4 (7.3–7.5)	0.73
Ammonia (venous; μmol/L)	38	94 (78–136)	17	75 (60–126)	0.23
Creatinine (mg/dL)	82	1.4 (0.8–3.1)	47	2.3 (1.0–3.8)	**0.049**
Lactate (mmol/L)	56	3.3 (2.1–6.3)	35	6.5 (3.9–11.0)	**0.003**
**Admitted to Intensive Care**	82	75 (91%)	48	47 (98%)	0.48
**NAC**	82	75 (91%)	48	44 (92%)	0.75
**Organ support (7-days)**					
Mechanical ventilation	82	45 (55%)	48	42 (88%)	<0.001
Vasopressors	82	8 (9.8%)	48	32 (67%)	<0.001
Renal Replacement therapy	82	21 (26%)	48	30 (63%)	**<0.001**
**ICP therapies (7 days)**					
Mannitol	82	14 (17%)	48	19 (40%)	**0.006**
Hypertonic saline	82	6 (7.3%)	48	4 (8.3%)	**1.0**
Barbiturates	82	10 (12%)	48	9 (19%)	**0.33**
Hypothermia	82	8 (9.8%)	48	7 (15%)	**0.43**
Sedatives					
**ICP monitor**	81	9 (11%)	45	8 (18%)	0.29
HE Grade (median IQR)	82	2(1–4)	48	3(2–4)	0.17
**HE Grade III/IV**	82	39 (48%)	48	31 (65%)	0.26
**OA level detectable**					
**Early** (μmol/L)	80	12 (15%)	48	15 (31%)	0.034
**Late** (μmol/L)	82	9 (11%)	48	13 (27%)	0.018
**OA level**					
**Early** (μmol/L)	80	0.36(1.65)	48	0.48 (1.45)	0.69
**Late** (μmol/L)	82	0.37(1.53)	48	0.43 (0.92)	0.79

**Abbreviations**: INR, international normalized ratio; ALT, alanine aminotransferase; AST, aspartate aminotransferase

7-days: Values refer to therapies at any time during the 7-days of study.

During the first 7 study days, survivors were less likely to require mechanical ventilation (55% vs. 88%), vasopressor support (9.8% vs. 67%) or renal replacement therapy (26% vs. 63%, p< 0.001 for all comparisons). Survivors were less likely to receive mannitol to treat intracranial hypertension (ICH) (17% vs. 40%, p = 0.006) but not other ICH directed therapies. There were no significant differences in requirements for invasive intracranial pressure monitoring (11% vs. 18%), median worst hepatic coma grade (West Haven) (2(1–4) vs. 3(2–4)) or number of patients with worst coma grade III or IV (48% vs. 65%; p>0.15 for all comparisons).

**Fig 1:**
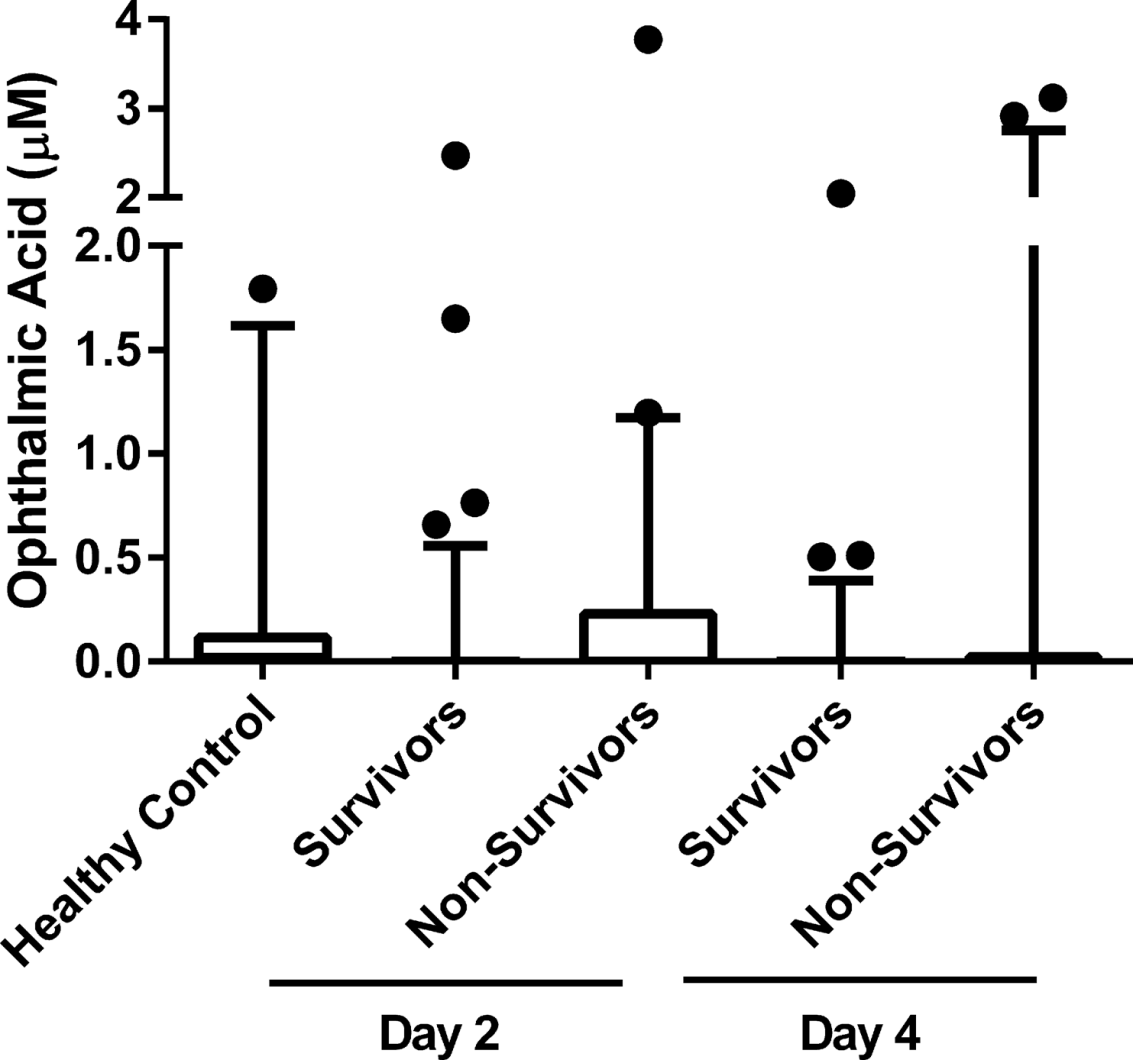
Ophthalmic acid levels in healthy controls compared with surviving and non-surviving APAP-induced ALF patients at Day 2 (early) and Day 4 (late). Blood samples were collected for 130 patients with APAP-induced ALF on Day 2 and Day 4 after admission into hospital; at Day 21, there were 82 survivors and 48 non-survivors. Serum OA levels were quantified using UPLC-MS-MS. The data are shown as a Box and Whiskers plot with boxes representing the interquartile range, lines representing the entire range, and data points representing the outliers. No statistically significant differences were found between groups using Chi-squared test.

Non-survivors were more likely to have detectable OA levels early (day 1 or 2: 31% vs. 15%, p = 0.034) and late (day 3 or 4; 27% vs. 11%, p = 0.018). However there were no statistically significant differences for overall mean levels between non-survivors and survivors (early 0.48 vs. 0.36, late 0.43 vs. 0.37, P > 0.5 for both) (Table 1). The range of detectable OA levels for the healthy controls, the survivors (early and late) and non-survivors (early and late) are shown in Fig 1. The lower limit of detection was 0.02 μM. Samples that fell below the limit of detection were set to 0 for data analysis. The mean OA level for the healthy human control group was 0.18 μM with a range from 0 μM to 1.18 μM. The survivors from both the early and late groups had a range of detectable OA levels from 0 μM to 2.47 μM and 0 μM to 4.09 μM respectively. OA levels were higher in non-survivors with a range of 0 μM to 3.77 μM and 0 μM to 3.12 μM in the early and late groups respectively, however statistical significance was not reached. This was also attributed to the fact that a large proportion of samples did not fall within the detectable range.

## Discussion

### Summary of key results

In this retrospective case control study of 130 APAP-ALF patients from the US ALFSG, OA was detectable more frequently in APAP-ALF non-survivors however OA was detected in only a minority of patients (~30%). Mean OA levels (early or late) were not predictive of outcome in APAP-ALF patients. These results might be explained by the use of therapeutic NAC increasing cellular cysteine levels allowing for GSH synthesis and reducing OA synthesis. Increased OA levels do not appear to predict outcome in APAP-ALF patients.

### Generalizability/Significance of Results

In this analysis, OA levels were detectable more frequently in APAP-ALF patients that died but not universally detected nor were mean levels correlated with outcome. We speculate that the lack of discriminatory power of OA in the human APAP-ALF setting is due to several reasons. Firstly, when patients intentionally overdose on APAP, they do not necessarily seek medical attention immediately after ingestion (some patients may present several days later). Given that the turnover rate of OA is rapid (peak serum levels in mice occurred 1 hour post-APAP dose with OA levels becoming similar to untreated animals after 6 hours) [6], the late presentation of patients after APAP overdose could result in reduced serum OA levels. Our results could imply that the use of NAC mitigates ongoing OA production. This is supported by the recent finding that OA levels are significantly elevated in patients with non-hepatic steatosis (who would not receive NAC therapy) [11]. There are also several confounding medical interventions in APAP-ALF patients that could potentially confound serum OA levels including dialysis/renal replacement therapy, oliguria/anuria, nutritional status and support (e.g. parenteral or enteral) and possibly other co-existent medical problems (e.g. bacteremia/sepsis).

These results are complementary to previous literature that shows NAC therapy increases cellular cysteine levels allowing for GSH synthesis and preventing OA synthesis. Previous well-documented clinical studies have demonstrated that NAC was beneficial in APAP-ALF through repletion of GSH, improved oxygen transport, improved hemodynamic status and reduced incidence of intracranial hypertension [12, 13]. Nonetheless, serum OA levels do not appear to yield clinically significant information regarding prognosis beyond traditional criteria based on hepatic synthetic activity (INR, Bilirubin, Factor V) [14, 15], severity of hepatic necrosis [16] or hepatic encephalopathy/coma grade.

### Study limitations

This study has several limitations that warrant consideration. It is a retrospective case control study which has potential bias due to selection of appropriate cases (non-survivors) and controls (survivors). We were unable to acquire matched numbers of APAP-survivors and non-survivors. While we did have healthy controls for comparison in our OA assay, we did not have access to serum samples from critically ill patients without ALF as controls. We do not believe that this would have had a significant impact on study conclusions. We did not have data on the exact timing of APAP ingestion for each patient compared to admission to hospital or amount ingested but practically speaking this is exceedingly difficult in clinical practice given that some patients may have had staggered APAP toxicity (i.e. not a single large ingestion but ongoing daily use of APAP). Finally, given that NAC is standard of care in suspected APAP toxicity, APAP-ALF, we did not have access to a significant number of patients with APAP-ALF in the absence of NAC. Despite these limitations, we believe this study has significant merit because to our knowledge it is the only study to examine serum OA levels in humans in APAP-ALF.

## Conclusions

OA was detectable more frequently in APAP-ALF non-survivors but mean OA levels were not associated with survival. The routine clinical administration of *N*-acetyl cysteine could diminish OA production and prevent OA from being a clinically useful biomarker in APAP-ALF.

### Key Points

- Ophthalmic acid (OA) is an analogue of GSH in which cysteine is replaced with 2-aminobutyrate.
- OA was detectable more frequently in acetaminophen-induced acute liver failure non-survivors
- Mean OA levels were not associated with survival.

## Supporting Information

S1 STROBE Checklist STROBE guideline for reporting retrospective studies. This file contains the **STROBE** guideline for reporting case control studies format used for the current study[17] (BMJ 2007).
(DOC)

## Abbreviations

ALF: acute liver failure
APAP: acetaminophen
HE: hepatic encephalopathy
GSH: glutathione
INR: international normalized ratio
MAP: mean arterial pressure (mm Hg)
MELD: model for end stage liver disease score
MV: mechanical ventilation
OA: ophthalmic acid
RRT: renal replacement therapy
UPLC-MS-MS: ultraperformance liquid chromatography tandem mass spectrometry
